# Identification of Methanogenic archaea in the Hyporheic Sediment of Sitka Stream

**DOI:** 10.1371/journal.pone.0080804

**Published:** 2013-11-20

**Authors:** Iva Buriánková, Lenka Brablcová, Václav Mach, Petr Dvořák, Prem Prashant Chaudhary, Martin Rulík

**Affiliations:** 1 Department of Ecology and Environmental Sciences - Laboratory of Aquatic Microbial Ecology, Faculty of Science, Palacky University, Olomouc, Olomouc, Czech Republic; 2 Department of Botany, Faculty of Science, Palacky University, Olomouc, Olomouc, Czech Republic; Argonne National Laboratory, United States of America

## Abstract

Methanogenic archaea produce methane as a metabolic product under anoxic conditions and they play a crucial role in the global methane cycle. In this study molecular diversity of methanogenic archaea in the hyporheic sediment of the lowland stream Sitka (Olomouc, Czech Republic) was analyzed by PCR amplification, cloning and sequencing analysis of the methyl coenzyme M reductase alpha subunit (*mcrA*) gene. Sequencing analysis of 60 clones revealed 24 different *mcrA* phylotypes from hyporheic sedimentary layers to a depth of 50 cm. Phylotypes were affiliated with *Methanomicrobiales, Methanosarcinales* and *Methanobacteriales* orders. Only one phylotype remains unclassified. The majority of the phylotypes showed higher affiliation with uncultured methanogens than with known methanogenic species. The presence of relatively rich assemblage of methanogenic archaea confirmed that methanogens may be an important component of hyporheic microbial communities and may affect CH_4_ cycling in rivers.

## Introduction

The decomposition of organic matter in aquatic sediments is an important process in global and local carbon budgets, as it ultimately recycles complex organic compounds from terrestrial and aquatic environments into carbon dioxide and methane. The latter is a major component in the carbon cycle of anaerobic aquatic systems. Since a relatively large amount of methane production has been observed in river sediments [1-4], we hypothesised that river sediments may act as a considerable source of methane gas emission into the environment [5]. 

Methane (CH_4_) is produced mostly by methanogenic archaea [6,7] as a final byproduct of anaerobic respiration and fermentation but there is also aerobic formation of methane by the aerobic degradation of methyl phosphonates [8] or by oxidation of ascorbic acid using iron compounds and hydrogen peroxide [9]. Methanogenic archaea belonging to the *Euryarchaeota* phylum are divided into seven orders: *Methanopyrales, Methanococcales, Methanobacteriales, Methanomicrobiales*, *Methanosarcinales* and the recently recognized groups *Methanocellales* and *Methanoplasmatales* [10,11]. Methanogenic archaea are ubiquitous in anoxic environments and require a redox potential of less than -300 mV for their growth [12]. They can be found in moderate habitats such as rice paddies [13], soils [14], lake sediments [15], in extreme conditions such as hydrothermal vents [16], permafrost soils [17,18] and also in the gastrointestinal tract of animals [19]. Freshwater sediments, including wetlands, rice paddies and lakes, are thought to contribute 40 to 50% of the annual atmospheric methane flux [20]. 

Rates of methane production and consumption in sediments are controlled by the relative availability of substrates for methanogenesis. The most important immediate precursors of methanogenesis are acetate and H_2_/CO_2_. The acetate is converted into CH_4_ and CO_2_ by acetoclastic methanogens while hydrogenotrophic methanogens convert CO_2_ and H_2_ or formate to CH_4_ [21]. Acetate is consumed by a limited number of strains such as *Methanosarcina* spp. and *Methanosaeta* spp., the latter are incapable of using hydrogen. A large quantity of the acetate is produced in natural ecosystems and acetoclastic methanogens are responsible for 30–70% of methane production from freshwater sediments [22]. Hydrogenotrophic methanogens of the genus *Methanobacterium*, are important in maintaining low levels of atmospheric H_2_ [23]. According to Conrad et al. [24], the degradation pathway of polysaccharides in methanogenic sediments (lakes, bogs, paddy fields, marine etc) is such that about two-thirds of the produced CH_4_ is theoretically derived from acetate and one third from H_2_/CO_2,_ if steady state conditions exist. The universal distribution of the hydrogenotrophic pathway suggests that hydrogenotrophic methanogenesis may be the ancestral form of biological methane production and that hydrogenotrophic methanogenesis appears only once in evolution [25]. Some studies showed that temperature conditions can be helpful for defining the structure and function of the methanogenic microbial community. Noll et al. [26] observed functional changes in rice fields soil from a mixture of acetoclastic and hydrogenotrophic methanogenesis to exclusively hydrogenotrophic methanogenesis over a temperature range of 42–46°C. Another study indicated that at 30°C, the methanogenic community in soil consists mainly of *Methanosarcinaceae*, whereas at 15°C, the diversity of methanogenic archaea is greater and includes for example members of the *Methanosaetaceae* family [27]. However, despite the fact that anaerobic metabolism is described in many lakes, estuaries and wetland sediments, there is a paucity of information on the methanogen diversity in river ecosystems.

Methanogenic archaea express the enzyme methyl-coenzyme M reductase which catalyzes the terminal step in biogenic methane production [28,29]. This enzyme complex is present in methanogens and methane oxidizers, making it a suitable tool for specific detection of methanogens. Methyl-coenzyme M reductase (*mcr*) constitutes about 5-12% of methanogen cellular protein and has been resolved into three components - A, C, and a small cofactor B. Component C is thought to be the site for methyl reduction and it is composed of three subunits; α, β and γ which are coded for by *mcrA, mcrB*, and *mcrG* genes respectively [30]. The genomes of all methanogenic archaea encode at least one copy of the *mcrA* operon [28]. The gene coding for *mcr* has been the target for many molecular ecological studies of methanogens [30-32]. The *mcr* operon exists in two forms, *mcrA* and *mrtA* gene coding. The *mcrA* gene is thought to be present in all methanogens, while the *mrtA* gene has only been demonstrated in members of the orders *Methanobacteriales* and *Methanococcales* [33].

The aim of this study was to identify and investigate the distribution of methanogens in two sediment depths (0-25 cm and 25-50 cm sediment layer) using the functional gene marker (the α-subunit of the methyl-coenzyme M reductase – *mcrA gene*). We used PCR, cloning and sequencing analysis for the determination of the methanogenic phylogenetic composition. In addition, analysis of dissolved methane, total cell numbers, abundance of methanogens and potential methane production were also measured at the chosen locality simultaneously. The results are part of a long-term study of organic carbon dynamics and associated microbial communities in hyporheic sediments of the small lowland Sitka stream in Olomouc, a city in the Czech Republic. Earlier measurements of relatively high methane production confirmed the suitability of the field site for the study of methane cycling [1,34].

## Materials and Methods

### Ethics statement

No specific permits were required for the described field studies. No permission was required for any locations or any activity. The locations are not privately owned or protected in any way. No activity during field study involved any endangered or protected species.

### Study site

The sampling sites are located on the Sitka stream, Olomouc, Czech Republic. Five study localities are placed along the Sitka stream and they were studied as a part of long term research on methanogenesis. Localities I and II are situated in an upper forested area. Localities III-V are situated in agricultural landscape. This study was on the locality IV, in particular. The Sitka is an undisturbed, third-order stream, 35 km long lowland stream originating in the Hrubý Jeseník mountains 650 m above sea level. The catchment area is 118.81 km^2^, the geology being composed mainly of Plio-Pleistocene clastic sediments of lake origin covered by quaternary sediments. The mean annual precipitation of the downstream part of the catchment area varies from 500 to 600 mm. Mean annual discharge is 0.81 m^3^s^-1^. The Sitka stream flows in its upper reach through a forested area with a low intensity of anthropogenic effects, while the lower course of the stream naturally meanders through an intensively managed agricultural landscape. Except for short stretches, the Sitka stream is unregulated with well-established riparian vegetation. River bed sediments are composed of gravels in the upper parts of the stream (median grain size 13 mm) while the lower part is several kilometres away from the confluence and is characterised by finer sediment with a median grain size of 2.8 mm. More detailed characteristics of the geology, gravel bar, longitudinal physicochemical (e.g. temperature, pH, redox, conductivity, O_2_, CH_4_, NO_3_^-^, SO_4_^2-^) patterns in the sediments and a schematic view of the site with sampling point positions have been published [34,35]. Earlier measurements of a relatively high production of methane, as well as potential methanogenesis, confirmed the suitability of the field sites for the study of methane cycling [1,5,34].

### Collection and processing of sediment sample

Based on the conclusions of the previous research carried on the Sitka stream in 2009 [36], locality no. IV was chosen from five localities for sampling sediment and interstitial water. The values of selected physico-chemical parameters of five localities, used in this study (Table 1), were the annual mean values of the reading collected during the year 2009 to 2011. Locality no. IV shows long- term extreme values in most cases (organic carbon in sediment, dissolved ferrous iron, acetate and methanogenic potential) (Table 1) and was chosen for more detailed study of the dynamics of methane and vertical distribution of methanogens. All samples were collected and parameters measured at 4th Oct 2010 (Table 2). The mean physico-chemical parameters and SDs were calculated for values of upper sediment layers (0-25cm) and deeper sediment layers (25-50 cm). Hyporheic sediments were collected with a freeze-core using liquid N_2_ as a coolant [37]. Altogether, three cores were gathered and taken for subsequent analyses. After sampling two layers, the surface 0-25 cm and 25-50 cm depth were immediately separated and stored at low temperature during transport to the laboratory. Immediately following thawing the wet sediment of each layer was sieved and only particles < 1 mm were considered for the following molecular analyses since most of the biofilm is associated with this fraction [38]. Four randomly selected subsamples (1 mL) from each core were used for the extraction of microbial cells and subsequently used for the estimation of bacterial and archaeal numbers. Four other subsamples were used for DNA extraction. 

**Table 1:** Selected physico-chemical parameters (annual means 2009-2010) of the hyporheic interstitial water and sediment along the longitudinal stream profile (average ± SD).

**Variable/ Locality**	**I**	**II**	**III**	**IV**	**V**
Organic carbon in sediment < 1 mm [%]	0.98 ± 0.12	0.91 ± 0.02	0.57 ± 0.31	1.31 ± 0.63	0.74 ± 0.30
Dissolved oxygen saturation [%]	80.48 ± 6.65	88.01 ± 2.91	82.38 ± 5.66	38.45 ± 29.37	50.91 ± 24.66
Ferrous iron Fe^2+^ [mg L^-1^]	< 1	< 1	1.78 ± 0.15	8.08 ± 5.76	4.23 ± 4.01
Acetate [mmol L^-1^]	0.21 ± 0.13	0.34 ± 0.18	0.52 ± 0.17	1.87 ± 0.55	0.29 ± 0.17
Dissolved methane concentration [µg L^-1^]	4.94 ± 3.45	0.71 ± 0.15	8.06 ± 1.65	2 480.19 ± 1145.10	42.83 ± 32.11
Methanogenic potential [pmol CH_4_ g^-1^ DW h^-1^]	1.73 ± 1.70	0.45 ± 0.02	0.53 ± 0.50	18.45 ± 25.16	1.71 ± 2.00
Interstitial water temperature [°C]	8.70 ± 0.85	9.44 ± 0.37	11.60 ± 1.27	11.20 ± 0.14	11.40 ± 3.53

**Table 2:** Vertical gradient of physico-chemical parameters and variables of the hyporheic interstitial water and sediment at the locality no. IV (average ± SD).

**Variable/ Depth**	**0-25 cm**	**25-50 cm**
Dissolved oxygen saturation [%]	59.37 ± 21.83	17.54 ± 6.02
Ferrous iron Fe^2+^ [mg L^-1^]	3.70 ± 3.98	12.26 ± 1.49
Acetate [mmol L^-1^]	1.72 ± 0.73	2.02 ± 0.12
Dissolved methane concentration [µg L^-1^]	2262.65 ± 2053.41	3856.01 ± 898.02
Methanogenic potential [pmol CH_4_ g^-1^ DW h^-1^]	191.5 ± 75.14	173,87 ± 61.84
Total cell number [10^6^ cells g^-1^DW]	4.28 ± 6.53	6.18 ± 6.13
Methanogens abundance [10^6^ cells g^-1^DW]	0.65 ± 1.21	1.01 ± 1.16

 Sediment organic matter content was determined by oven-drying at 105°C to constant weight and subsequent combustion at 550°C for 5 h to obtain ash-free dry weight (AFDW). Organic matter values were then converted to carbon equivalents assuming 45% carbon content of organic matter [39].

### Collection of water samples and methane analysis

Interstitial water samples were collected using a set of 5–6 minipiezometers [40] randomly placed into hyporheic sediments on locality IV at specified depths (Table 2, for more details see File S1). The initial 50–100 mL of water was used as a rinse and discarded. Two subsamples of interstitial water from each minipiezometer were then collected from a continuous column of water with a 100 mL polypropylene syringe connected to a stiff PVC tube. The subsamples were injected into separate sterile, clear vials (40 mL) with screw-tops, covered by a polypropylene cap with PTFE silicone septa (for analysis of dissolved gases) and stored before returning to the laboratory. All samples were taken in the morning and all measurements were done at base flow. Interstitial water temperature, dissolved oxygen (percent saturation) were measured in the field with a portable Hanna HI 9828 pH/ORP/EC/DO multimeter (Fischer Scientific, USA). Dissolved ferrous iron (Fe^2+^) concentration was measured using absorption spectrophotometry after reaction with 1, 10-phenanthroline. Concentrations of organic acids were measured using capillary electrophoresis equipped with diode array detector HP 3D CE Agilent (Waldbron, Germany). Limits of detection (LOD) for particular organic acids were set as follows: LOD (acetate) = 6.2 µmol L^-1^; LOD (propionate) = 4.8 µmol L^-1^; LOD (butyrate) = 2.9 µmol L^-1^; LOD (valerate) = 1.8 µmol L^-1^. 

Concentrations of dissolved methane in the interstitial water were measured directly using a headspace equilibration technique. Dissolved methane was extracted from the water by replacing 10 mL of water with N_2_ and then the vials were vigorously shaken for 15 s (to release the gas from the water to facilitate equilibration between the water and gas phases). All samples were equilibrated with air at room temperature. Methane was analysed from the headspace of the vials by injecting 2 mL of gas subsample with a gas-tight syringe into a CHROM 5 gas chromatograph, equipped with the flame ionization detector (CH_4_ detection limit = 1µg L^-1^) and with the 1.2 m PORAPAK Q column (I.D. 3 mm, Sigma-Aldrich, Germany), with nitrogen as a carrier gas. Gas concentration in water was calculated using Henry’s law. 

### Analysis of methanogenic potential

The rate of methane production (methanogenesis) was measured using the potential methane production method [41]. The sediment was sieved and placed in incubation flasks. C-amended solutions (flushed for 5 min with N_2_) with acetate Ca (CH_3_COO)_2_ (100 mg C in the incubation flask) were used for examination of the methanogenic potential. The substrate, acetate, was chosen as our earlier results for the same study site showed that acetate is more important than hydrogen for methanogenesis in hyporheic sediments [1]. All laboratory sediment incubations were performed in 250-mL dark glass flasks, capped with rubber stoppers, using approximately 100 g (wet mass) of sediment (grain size < 1 mm) and 180 mL of amended solution or distilled water. The headspace was maintained at 20 mL. Typically, triplicate live and dead samples (methanogenesis was inhibited by addition of 1.0 mmol chloroform) from each depth were stored at 20°C in the dark and the incubation time was 72 h; however, subsamples from the headspace atmosphere were taken every 24 h. Gas production was calculated from the difference between initial and final headspace concentration and volume of flask; results are expressed per unit dry weight of sediment per one hour (pmol CH_4_ g^-1^ DW h^-1^). 

### Abundance of microbial cells and methanogens

For measuring microbial parameters, paraformaldehyde fixed samples of sediment were sonicated followed by incubation with detergent mixture (Tween 20, 0.5% v/v) and density centrifugation (non-ionic medium Nycodenz, 1.31 g mL^-1^) was used. 

The supernatant was filtered onto membrane filter. The abundance of methanogenic archaea was identified by fluorescence *in* *situ* hybridization (FISH) with 16S rRNA-targeted methanogen-specific oligonucleotide probe MPB1 5’-CAT GCA CCW CCT CTC AGC -’3 [42] labelled with indocarbocyanine dye Cy3. The MPB1 probe was designed to target position 978–996 (*E.coli*). The prokaryotes were hybridized according to the protocol [43]. Staining with DAPI solution (6.3 mg mL^-1^; w/v) for total cell number (TCN) estimation was then used (more detailed protocol is described in [41]). Stained cells were enumerated on an epifluorescence microscope (Olympus BX 60, 1000 × magnification, Olympus corporation, Japan) equipped with a camera (Olympus DP 12) and image analysis software (NIS Elements; Laboratory Imaging, Prague, Czech Republic). At least 200 cells within at least 20 microscopic fields were counted in three replicates from each sediment layer. Total cell numbers (TCN) were expressed as cell numbers per 1 g of dry sediments.

### Nucleic acid extraction and PCR amplification

Nucleic acids were extracted from 0.5 g of sieved sediment with a Power Soil DNA isolation kit (MoBio, Carlsbad, USA) according to the manufacturer’s instructions. Fragments of the methanogenic DNA (~ 470 bp) were amplified by PCR using *mcrA* gene specific primers. Primer sequences for *mcrA* gene are as follows, 

*mcrA* F 5’-GGTGGTGTMGGATTCACACARTAYGCWACAGC-3‘, 

*mcrA* R 5’-TTCATTGCRTAGTTWGGRTAGTT-3‘

[29]. PCR amplification was carried out in a 50 µL reaction mixture within 0.2 mL thin walled micro-tubes. Amplification was performed in a TC-XP thermal cycler (Bioer Technology, Hangzhou, China). The reaction mixture contained 5 µL of 10 × PCR amplification buffer, 200 µmol of each dNTP, 0.8 µmol of each primer, 2 µL of template DNA and 2.5 U of FastStart Taq DNA polymerase (Polymerase dNTPack; Roche, Mannheim, Germany).

The initial enzyme activation and DNA denaturation were performed for 6 min at 95°C, followed by 5 cycles of 30 s at 95°C, 30 s at 55°C and 30 s at 72°C, and the temperature ramp rate between the annealing and extension segment was set to 0.1°C/s because of the degeneracy of the primers. The ramp rate was then set to 1°C/s, and 30 cycles were performed under the following conditions: 30 s at 95°C, 30 s at 55°C, 30 s at 72°C and a final extension at 72°C for 8 min – according to the protocol [44]. PCR products were visualised by horizontal gel electrophoresis in ethidium bromide stained, 1.5% (w/v) agarose gel.

### Cloning, sequencing and phylogenetic analysis

Purified PCR amplicons (PCR purification kit; Qiagen, Venlo, Netherlands) were ligated into TOPO TA cloning vectors and transformed into chemically competent *Escherichia coli* TOP10F’ cells according to the manufacturer’s instructions (Invitrogen, Carlsbad, USA). Positive colonies were screened by PCR amplification with the primer set and PCR conditions as described above. Plasmids were extracted using UltraClean 6 Minute Plasmid Prep Kit (MoBio, Carlsbad, USA), and nucleotide sequences of cloned genes were determined by sequencing with M13 primers in Macrogen company (Seoul, Korea).

Raw sequence data were then analyzed by BLAST software to search for the similarity with other methanogen sequences available in the GenBank database. The sequences were then aligned by using CLUSTAL W [45] software in order to remove any chimeric sequences. The most appropriate substitution model for maximum likelihood analysis was identified by Bayesian Information Criterion implemented in MEGA 5.05 software [46]. The phylogenetic tree was constructed by the maximum likelihood method (Kimura 2-parameter model, gamma variation across sites). The tree topology was statistically evaluated by 1000 bootstrap replicates (maximum likelihood) and 1000 bootstrap replicates [47]. The cut-off value for determination of identical sequences was 97%.

GenBank accession numbers for methanogenic *mcrA* gene sequences retrieved from Sitka are as follows: 

KC952027-KC952036, KC952039, KC952041-KC952042, KC952043-KC952048, KC952050-KC952052 and KF156778- KF156780.

### Statistics

The analyses were performed using statistical software R version 2.6.0. Studied parameters were analyzed by a Wilcoxon signed rank sum test. All tests were considered significant at probability level p < 0.05.

## Results and Discussion

### Environmental and microbial parameters of the river hyporheic sediment

Generally, interstitial water revealed relatively high dissolved oxygen saturation with the exceptions of localities V and IV where the concentration of dissolved oxygen sharply decreased with depth. However, it never dropped below ∼ 10% of the oxygen saturation (data not shown). Vice versa, these two localities were also characterized by much higher concentrations of dissolved ferrous iron and dissolved methane than sites located upstream (Table 1). Concentration of ferrous iron reflected the anaerobic conditions of the interstitial environment and showed the highest concentration in the deepest sediment layer (25-50cm) (p=0.02; n=5) at locality IV (Table 2). In aquatic environments, iron can be found in two forms. In oxic environments iron occurs in the form of ferric iron (Fe^3+^) and in anoxic enviroment ferrous iron (Fe^2+^) dominates. Ferric iron is reduced to ferrous iron in anoxic conditions by bacteria which utilize this reduction process for their growth. This reduction can be carried out using one of several reductans such as hydrogen, pyruvate, lactate, acetate etc. [48].

 Localities IV and V are situated in the lowland part of the stream which naturally meanders through an intensively managed agricultural landscape with increasing trophic level in the environment. The river bed sediment in lowland parts is characterised by fine sediment with organic matter accumulation. These budgets of organic matter allow local anoxic conditions. Locality IV, in particular, showed high concentration of organic carbon, ferrous iron and acetate in the sediment. These parameters facilitate the presence of methanogens in sediment and this is supported by the high concentration of dissolved methane in intestitial water and the methanogenic potential measurements (Table 1). 

The average annual temperature of interstitial water at localities in the downstream parts of the Sitka stream was about 2.5°C higher than in localities upstream and this may result in higher methane production in the region. 

The precursor of methanogenesis, acetate was found in the interstitial water at all study sites and measured regularly at higher concentration with maximum concentration usually during the summer period (Table 2). However, the concentration of other precursors such as propionate, valerate and butyrate were also measured but the values were under detection limits (data not shown).

At locality no. IV the mean methane concentration in the interstitial water ranged between 2262.65 - 3856.01 µg L^-1^ at 0-25 and 25-50 cm depths, respectively (Table 2). However the differences were not significant (p=0.11; n=5). Generally, the methanogenic potential (MP) varied around 180 pmol CH_4_ g^-1^ DW h^-1^ (0,21 nmol CH_4_.g.WW.h^-1^, respectively) at the study site. The methanogenic potential was found to be similar in both sediment layers (p=0.82; n=5) (Table 2). These results show decreased readings compared to our previous study [34], in which a considerable amount of methane production was found in the upper sediment layer. To date, there is no standardised approach to measuring potential methane production and hence different results could be due to different methodologies. The MP range is quite broad and may differ by up to three orders of magnitude (10^– 2^ to 10^1^ µmol m^-3^ s^-1^,, 10^3^-10^6^ nmol m^-3^ h^-1^ respectively); however, depending on temperature or availability of electrons, it can reach up to 10^8^ nmol m^-3^ h^-1^. Increasing temperature apparently raises the ability to produce methane [41]. Study of anaerobic incubation of sediment acceptors for methanogens of chalk streams revealed maximum MP at a depth of 6 cm (16.5 CH_4_ nmol g^-1^ wet sed. h^-1^) with MP decrease with increasing sediment depth. However these authors only investigated a depth 0-8 cm under the river bed [2]. 

Although there was found a lot of variability in interstitial methane concentration and methanogenic potential, the values of both the parametres suggest that the studied locality (no. IV) produces a lot of methane and hence would be suitable for analysis of methanogen diversity in the region. Fluctuation in the data even during measurements conducted at the same day should not be suprising when considering the very dynamic system of hyporheic zone. Concentrations of methane may differ by up to several orders of magnitude both in horizontal and vertical profile of the sediments [49].

Total prokaryote cell numbers showed a significantly higher value in the deeper sediment layer (6.18×10^6^ cells g^-1^DW) than in the upper sediment layer (4.28×10^6^ cells g^-1^DW) (p=0.02; n=15). The abundance of methanogenic archaea identified with probe MPB1 was higher in deeper sediment layer, however the values were not significantly different (p=0.06; n=15) (Table 2). The values of the total cell numbers obtained from river sediment were relatively low compared to that of other sediments which varied from 10^6^ - 10^10^ per g^-1^DW [50,51]. This could be explained by the use of density centrifugation and sonication which can potentially damage prokaryote communities and influence total cell numbers and diversity. 

However, additional purification of cells by a combination of sonication and detergent treatment, followed by density gradient centrifugation is often recommended for soil, sediment or biofilm samples [52]. Even if the total cell numbers are slightly underestimated, sonication and density centrifugation techniques are still powerful enough to enable comparison between equally treated samples [53]. Further, checking of direct microscopic cell counts after sonication showed that the efficiency of the sonication was 85–90% (as shown in our previous experiments). Direct counting of bacteria in sediment is limited due to masking of bacteria by sediment particles. Density centrifugation results in the separation of bacteria from sediment particles and improves the purity of cell suspensions [54].

### Identification of methanogens based on mcrA genes

**Figure 1:**
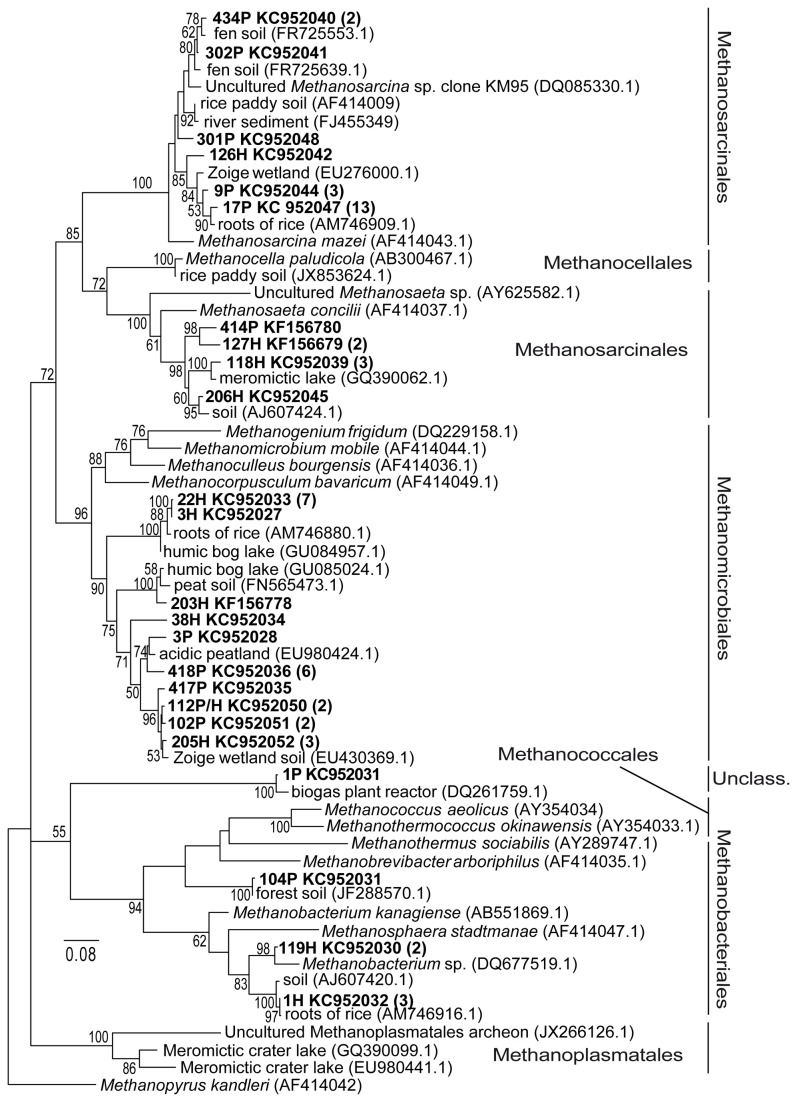
Phylogenetic tree of *mcrA* gene clone (phylotype) sequences retrieved from hyporheic river sediment. The clones come from upper sediment layer (0-25 cm depth) are described as „P“, clones come from deeper sediment layer (25-50 cm depth) are described as „H“. The numbers in parenthesis indicate the number of clones. The phylogenetic tree is rooted with *Methanopyrus kandleri*.

The methanogenic community in the hyporheic sediment of the Sitka stream was also analyzed by PCR amplification, cloning, and sequencing of the methyl coenzyme M reductase (*mcrA*) gene. A total of 60 *mcrA* gene sequences revealed 24 different phylotypes. These phylotypes were clustered into four groups and they confirmed affiliation to *Methanosarcinales* (10 phylotypes), *Methanomicrobiales* (10 phylotypes, one phylotype was found in both sediment layer, respectively) and *Methanobacteriales* orders (3 phylotypes) and uncultutured group (1 phylotype) of methanogens. Six members of the *Methanosarcinales* order were affiliated to the *Methanosarcinaceae* family and the nearest identical uncultured sequences were obtained from fen soil in Germany [55], Tibetian Zoige wetland sediment [56] and rice roots grown in Holland [57]. Four phylotypes were related to the acetoclastic methanogen *Methanosaeta concilii* and showed the highest similarity with sequences retrieved in a meromictic lake sediment in France [58] and flooding soil in Holland [59]. Within the *Methanomicrobiales* order, ten different phylotypes were detected and clustered along with the uncultured sequences, which were obtained from humic bog lake [60], acidic peatland [61], peat soil from Finland [62], Tibetian wetland soil [56] and rice roots in Holland [57]. Three phylotypes were related to the *Methanobacteriales* order and of these three phylotypes, one was closely affiliated to *Methanobacterium* sp. which was isolated from a Western Siberian peat bog [63] and the second one was clustered with clone originating in flooding soil [59] and rice root samples in Holland [57]. One single clone remains unclassified and was clustered with a sequence, which came from a biogas plant reactor in India [64]. It however showed 76% identity with *Methanobacterium* sp. Most of the clones assigned in this study showed low affiliation with known methanogenic species and were closely related to uncultured methanogens obtained from other similar environments (Figure 1). Some environmental studies also confirmed that some clones may constitute an unclassified methanogenic cluster [56–58]. 

Members of all three orders were detected in a whole bottom sediment irrespective of depth. The number of clones affiliated with *Methanomicrobiales* predominated in the deeper layer while numbers of *Methanosarcinales* clones in general were higher in the upper sediment layer. However, higher number of *Methanosarcinaceae*-like clones were found in the upper layer and *Methanosaetaceae*-like clones prevalenced in the deeper layer of sediment (Table 3). 

**Table 3:** Number of clones and phylotypes and their phylogenetic affiliation to each library.

**Variable/ Depth**				
Phylogenetic affiliation	Methanomicrobiales	Methanosarcinales (Methanosarcinaceae/ Methanosaetaceae)	Methanobacteriales	unclassified
No. of clones				
[depth 0-25 cm]	11	21 (20/1)	1	1
[depth 25-50 cm]	14	7 (1/6)	5	0
No. of phylotypes				
[depth 0-25 cm]	5	6 (5/1)	1	1
[depth 25-50 cm]	6	4 (1/3)	2	0
Clones occurrence [%]				
[depth 0-25 cm]	32	59/3	3	3
[depth 25-50 cm]	54	4/23	19	0

The coverage (C) of each clone library, a measure of captured diversity, was calculated as: C=1-(n/N), where *n* is the number of different phylotypes from a clone library that were encountered only once and *N* is the total number of sequenced clones in the library [65]. The coverage of each library was 76,5% for upper sediment layer (0-25 cm) and 76,9% for deeper sediment layer (25-50 cm). 

Of the total number of 24 methanogenic phylotypes identified from hyporheic sediment, 13 phylotypes (34 clones, 57% from total number of clones) were found in the upper sediment layer (0-25 cm). One phylotype obtained from the upper layer (1 clone, 3%) was affiliated to the *Methanobacteriales*. Phylotypes related to the *Methanosarcinales* order including 5 phylotypes (20 clones, 59%) *Methanosarcinaceae*-like and one phylotype (1 clone, 3%) *Methanosaetaceae*-like member. Larger number of *Methanosarcinales*-like archaea in the upper sediment layer was also confirmed by FISH analyses (unpublished data). Five phylotypes (11 clones, 32%) retrieved from the upper layer were related to the *Methanomicrobiales*. A single unclassified methanogen clone (3%) was retrieved from the upper sediment layer. 

Twelve phylotypes (26 clones, 43% from total number of clones) were detected in the deeper sediment layer (25-50 cm). Six phylotypes (14 clones, 54%) were related to the *Methanomicrobiales*. Four phylotypes (7 clones, 27%) were affiliated to the *Methanosarcinales* order, *Methanosarcinaceae*-like member including one phylotype (1 clone, 4%) and *Methanosaetaceae*-like member consists of three phylotypes (6 clones, 23%). Two phylotypes (5 clones, 19%) of these were associated with the genus *Methanobacterium*, 

Our results indicate the presence of both hydrogenotrophic and acetoclastic methanogens in river sediment. These observations are supported by the stable carbon isotope signature of methane (δ^13^CH_4_) which shows that both acetoclastic and hydrogenotrophic pathways take part in methanogenesis along the vertical profile of the Sitka stream [66]. The latest results show that the acetoclastic pathway predominates over the hydrogenotrophic pathway in a whole bottom sediment irrespective of depth and contributes to the methanogenesis in the Sitka stream with approximately 70 - 80% (unpublished data). 

*Methanomicrobiales* group of methanogens only grow in the presence of hydrogen, formate and alcohols with the exception of methanol. The *Methanosarcinaceae* can utilize all methanogenic substrates except for formate but the *Methanosaetaceae* grow exclusively using acetate as an energy source [67] whereas *Methanobacteriales* grow by CO_2_ reduction. In the Sitka stream sediment, the number of phylotypes related to *Methanomicrobiales* and *Methanosarcinales* was equivalent. However the number of *Methanosarcinales* clones (n=28) was higher over *Methanomicrobiales* clones (n=25). As suggested in an earlier study [68], members of these two orders may be efficient syntrophic partners in the complete degradation of organic biomass in freshwater sediments. Only one study mentions methanogens in river sediment. This research investigated microbial populations in the extremely metal-contaminated Coeur d'Alene River sediments but the authors found just three methanogen phylotypes related to the *Methanosarcinales* order [69]. Most of the earlier reports on methanogens diversity were came from ruminants [70–72].

Moreover, DGGE analyses based on 16S rDNA of the methanogen community of the Sitka stream hyporheic sediments also retrieved a resembling number of taxonomic units at locality no. IV and this supports the results of this study (unpublished data). 

The application of PCR-based technologies for the investigation of naturally occurring methanogen populations has several advantages [72]. These methods are effective for detecting novel sequences, indicating unculturable new species and providing more complete description of the methanogen community structure. However, molecular methods introduce their own bias, such as the favoured lysis of one cell type over another, leading to the recovery of unrepresentative DNA fractions or skewed PCR amplification, where certain bands are favoured over others [33]. Another form of PCR bias is template reannealing which may occur during PCR when a high concentration of a product has accumulated and similar products and templates reanneal to each other, inhibiting primer binding and further amplification of a product [73]. It has also been suggested that the variability in copy numbers and intraspecies and interspecies heterogeneity of functional genes may represent a source of biases in microbial ecological studies [74]. 

## Conclusion

To the best of our knowledge, this study is the first analysis of the methanogenic community composition in river hyporheic sediments with respect to the process of methanogenesis. The presence of methanogenic archaea was detected using *mcrA* gene marker and FISH (MPB-1 oligonucletide probe) to a 50 cm river sediment depth. The data from the *mcrA* gene sequencing, retrieved a relatively large number of methanogenic phylotypes. These results support our previous measurements and suggest that methanogens contributes significantly to the hyporheic microbial community and may affect CH_4_ cycling in the Sitka stream sediments. The results also indicate the presence of both hydrogenotrophic and acetoclastic metabolic pathways in the Sitka river sediment. We hope these findings will be helpful for further research on the ecological function of methanogens in the carbon cycle in river hyporheic sediments. 

## Supporting Information

File S1 **Original data of physico-chemical parameters and variables of the hyporheic interstitial water and sediment used for Table 2 calculation.**
(XLS)
